# Tularemia Outbreak Investigation in Kosovo: Case Control and Environmental Studies

**DOI:** 10.3201/eid0801.010131

**Published:** 2002-01

**Authors:** Ralf Reintjes, Isuf Dedushaj, Ardiana Gjini, Tine Rikke Jorgensen, Benvon Cotter, Alfons Lieftucht, Fortunato D’Ancona, David T. Dennis, Michael A. Kosoy, Gjyle Mulliqi-Osmani, Roland Grunow, Ariana Kalaveshi, Luljeta Gashi, Isme Humolli

**Affiliations:** * Institute of Public Health North Rhine-Westphalia, Munich, Germany; † Institute of Public Health, Pristina, Kosovo; ‡ World Health Organization, Pristina, Kosovo; § World Health Organization Regional Office for Europe, Copenhagen, Denmark; § Istituto Superiore di Sanita, Rome, Italy; ¶ #European Programme for Intervention Epidemiology Training, Paris, France; ** PHLS Communicable Disease Surveillance Centre, London, United Kingdom; †† Centers for Disease Control and Prevention, Atlanta, Georgia, USA; ‡‡ German Reference Laboratory on Tularemia, Munich, Germany

**Keywords:** tularemia, outbreak investigation, epidemiology

## Abstract

A large outbreak of tularemia occurred in Kosovo in the early postwar period, 1999-2000. Epidemiologic and environmental investigations were conducted to identify sources of infection, modes of transmission, and household risk factors. Case and control status was verified by enzyme-linked immunosorbent assay, Western blot, and microagglutination assay. A total of 327 serologically confirmed cases of tularemia pharyngitis and cervical lymphadenitis were identified in 21 of 29 Kosovo municipalities. Matched analysis of 46 case households and 76 control households suggested that infection was transmitted through contaminated food or water and that the source of infection was rodents. Environmental circumstances in war-torn Kosovo led to epizootic rodent tularemia and its spread to resettled rural populations living under circumstances of substandard housing, hygiene, and sanitation.

Kosovo (pop. approximately 2 million) has undergone severe social and economic disruption. By mid-1999, more than 10 years of political crisis and warfare had resulted in environmental disruption, mass population displacements, and a breakdown of sanitation and hygiene. Many essential public health functions, such as disease surveillance and outbreak response, had collapsed. In January 2000, the Kosovo Institute of Public Health (IPH) in Pristina implemented a new surveillance system for 20 communicable disease syndromes. On March 22, 2000, a public health physician in western Kosovo reported a cluster of patients with an unusual syndrome of fever, pharyngitis, and pronounced cervical lymphadenitis. Tularemia was clinically suspected, and the diagnosis was serologically confirmed on April 14 at the World Health Organization (WHO) regional reference laboratory in Rome (*1*). Active case-finding identified other patients at multiple sites in Kosovo who in the previous 6 months had a similar syndrome. Public health records as far back as 1946 disclosed no prior reports of tularemia in Kosovo, but the disease has been reported, although infrequently, from other areas of Yugoslavia and other Balkan states (*2*).

Tularemia, a zoonotic disease caused by the highly infective, virulent, nonsporulating gram-negative coccobacillus *Francisella tularensis*, is found throughout most of the Northern Hemisphere in a wide range of animal reservoir hosts. In addition, the organism can be isolated from contaminated environmental sources such as water and mud. It is transmitted to humans by various modes, including direct handling of infectious carcasses, ingestion of contaminated food or water, arthropod bites, or inhalation of infectious dusts or aerosols. Person-to-person transmission is not known to occur. Epidemics can often be traced to concurrent epizootics involving rodents and lagomorphs (rabbits and hares) (*3*,*4*), which may be associated with unusual increases in population density of these animal hosts. Generalized symptoms of tularemia typically have sudden onset, often with high fever, chills, fatigue, headache, pharyngitis, sore joints, chest discomfort, dry cough, vomiting, abdominal pain, and diarrhea. The usual incubation period is 3 to 5 days, although it can be as long as 21 days.

In humans, there are several tularemia syndromes, mostly depending on the portal of infection. Ingestion typically results in oropharyngeal tularemia, with fever, pharyngitis, cervical lymphadenitis, and suppuration (Figure 1). Other tularemia syndromes include ulceroglandular, glandular, pleuropneumonic, and systemic (typhoidal) forms (*5*). Severity of illness is related to biotype: *F. tularensis* biovar palaearctica (Type B), which causes tularemia in Europe, is less virulent than *F. tularensis* biovar tularensis (Type A), which is associated with sometimes severe and fatal illness in North America (*6*). Without antimicrobial treatment, tularemia can be acute and fulminant or protracted and debilitating.

**Figure 1:**
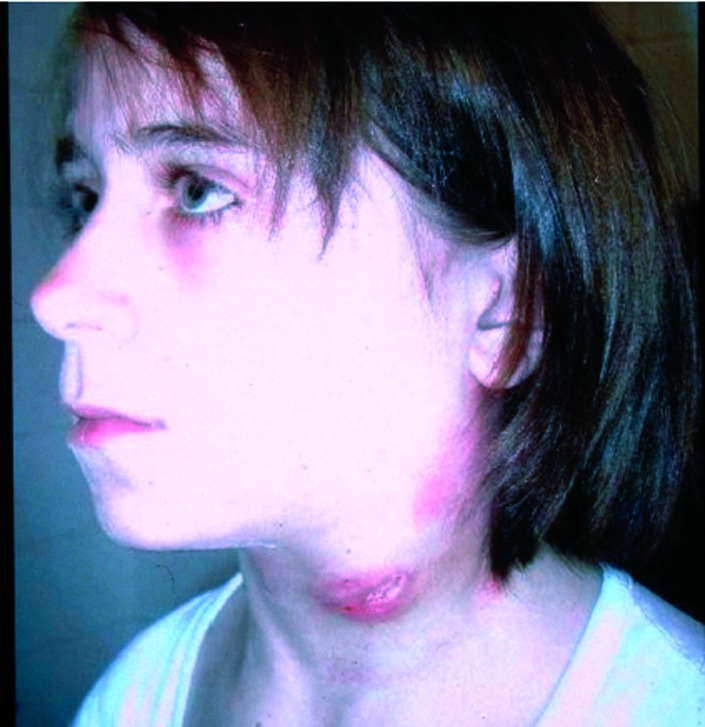
Girl with ulcerating lymphadenitis colli due to tularemia, Kosovo, April 2000.

Suspecting a widespread outbreak of tularemia in Kosovo, on April 14, 2000, the United Nations Mission in Kosovo requested WHO to assist Kosovar health authorities in an epidemiologic investigation. Teams of international and Kosovar public health personnel collaborated in field and laboratory investigations of epidemiologic, environmental, and microbiologic factors. The aims were to identify infection sources, vehicles, and modes of transmission and recommend appropriate control measures. In this report, we describe a case-control study of household risk factors and supporting environmental observations.

## Methods

###  Surveillance

In early April 2000, local authorities initiated active tularemia case finding by enhanced retrospective and prospective surveillance. Hospitals and clinics in Kosovo were asked to report all cases of illness with fever, lymphadenitis, and skin ulceration since the summer of 1999. Particular attention was paid to a syndrome of pharyngitis and cervical lymphadenitis. Investigators conducted in-depth interviews of persons with suspected cases during visits to homes. In addition, these ill persons were clinically examined and interviewed; if the illness was presumed to be tularemia, blood samples were drawn for serology and patients were treated with antibiotics, as appropriate. During the interviews, rural villagers frequently reported that the numbers of mice and rats had exploded in the spring and summer of 1999. A causal association between increased population density of rodents and human tularemia was hypothesized, based on suspected contamination of the environment by *F. tularensis*-infected rodents.

### Case-Control Study

**Figure 2:**
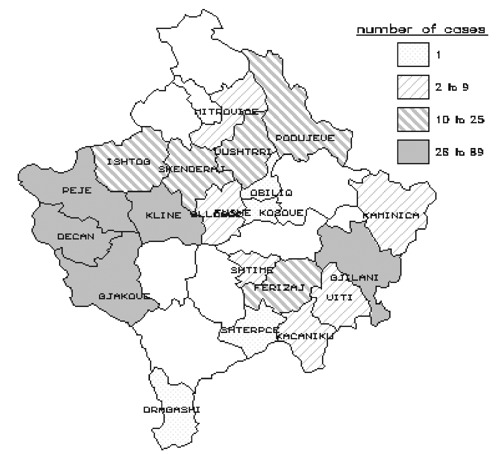
Total number of confirmed tularemia cases in Kosovo by municipality, July 1999-May 2000. Unshaded areas are Serb minority municipalities from which no data were available.

To test the hypothesis that a rodent-contaminated environment was the source of human tularemia and to determine household risk factors for infection, a matched case-control study was conducted with paired households as study units. Case households were defined as households with one or more family members having a laboratory-confirmed case of tularemia since November 1, 1999. Control households were defined as the two households closest to a case household, with no family member having a history of a syndrome of fever, pharyngitis, and cervical lymphadenitis since November 1, 1999, and with the person who prepared the family’s food being serologically negative for tularemia. Blood specimens were drawn from all persons with suspected cases, and in the control households, blood was drawn from the person responsible for preparing the household food. Structured questionnaires were completed on household food consumption, water supply, and presence of rodents in fields and domestic environments, and observations were recorded on conditions of wells and food preparation and storage areas. The study period began a month before onset of symptoms of the first case in the case household. The case and control household investigations were conducted in small villages in rural farming areas in Peje, Istog, Kline, and Deqan municipalities in the Peje Region, and in several villages in the adjacent Gjakova Region, western Kosovo (Figure 2). These regions were selected for study because they had the greatest numbers of reported cases.

Bivariate statistical analyses were performed in a matched pair analysis by chi-square test and Fisher’s exact test. Odds ratios (OR) (95% confidence intervals [CI]) were calculated by Epi Info 6.04 (*7*). Multivariate analyses were performed by conditional logistical regression by using a backward elimination model with Logistics (*8*).

### Environmental Investigations

Rodents were collected by baited live traps placed in homes, outbuildings, gardens, and fields of selected case households and neighboring households. Captured rodents were euthanized and identified to species, and blood, liver, and spleen specimens were collected. Blood samples were kept refrigerated and spun within 24 hours for separation of serum. Homes, outbuildings, dug wells, and other water sources were examined for evidence of contamination by rodent carcasses or excrement. Several rodent carcasses were collected from wells. Fecal specimens were collected from captured animals and from food storage and preparation areas of case and neighboring households. Water samples were collected from some wells and streams, and mud samples were collected from well heads and streams.

Serologic testing was done for immunoglobulin (Ig) M, IgG, and IgA isotype-specific antibodies by enzyme-linked immunosorbent assay (ELISA), microagglutination assay for screening, and Western blot for confirmation (*9*). Rodent tissues and feces were tested for *F. tularensis* lipopolysaccharide (LPS) by antigen-capture ELISA (*10*). Growth of *F. tularensis* on culture media was not attempted.

## Results

### Descriptive Epidemiology

**Figure 3:**
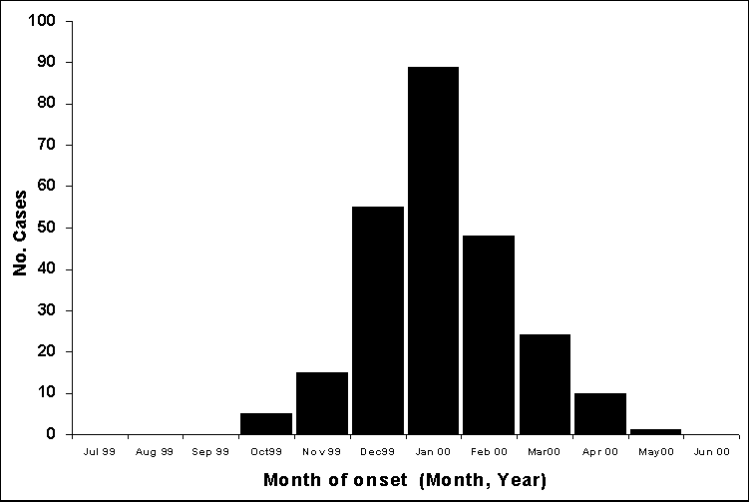
Epidemic curve of laboratory-confirmed tularemia cases (n = 247) in Kosovo, by month of onset of symptoms, October 1999- May 2000.

By June 30, 2000, >900 suspected cases of tularemia had been identified by IPH. This total included cases identified by retrospective record review, as well as prospectively by hospitals and clinics and by health teams conducting village surveys. Of these ill cases, 912 had serologic examinations and 327 were confirmed as tularemia positive. The earliest onset of reported symptoms in any of the confirmed cases occurred in October 1999. The epidemic curve of confirmed cases shows a peak in January 2000 (Figure 3). Confirmed cases were identified in 21 of 29 municipalities in Kosovo. (Several municipalities under Serbian authority did not submit data to IPH Kosovo; however, no cases from these areas were reported to IPH Belgrade [unpub. data, WHO, Belgrade]). Most cases were found in the western part of Kosovo (Figure 2), almost all from rural areas. Cases were equally distributed by sex (female 51.8%; male 48.2%), and all age groups were affected (median age 18 years; range <1 to 76 years).

### Analytical Epidemiology

To test the principal hypothesis that tularemia was associated with the consumption of food or water contaminated by *F. tularensis*-infected rodents, 46 case households and 76 control households were included in a matched analysis. Visual inspection of the case and control households confirmed the similarity of environmental and socioeconomic living conditions. Bivariate matched pair analyses of questionnaire data indicated that members of case households were less likely to have eaten fresh vegetables than were control households (Table 1). Case households were more likely to have water sources (mostly crude, open wells) unprotected against rodents (Table 1). Conditional logistic regression modeling indicated these independent risk factors for a case household: rodent feces in food preparation and storage areas (OR 5.7; 95% CI 1.5-22.2 [Table 2]) and large numbers of field mice seen outside the house (OR 5.6; 95% CI 1.1-28.7). The greater odds ratio for observing field mice obtained by multivariate analysis compared with bivariate analysis suggests either interaction of terms in the regression model or confounding in the bivariate analysis, although we were unable to identify either. Two factors were independently protective: household water source protected against rodents (OR 0.2; 95% CI 0.02-0.99) and eating fresh vegetables (OR 0.1; 95% CI 0.01-0.8) (Table 2).

**Table 1:** Household risk factors for tularemia determined by bivariate matched comparison of 46 case households and 76 control households, Kosovo, October 1999-May 2000

Risk factor	OR^a^	95% CI	p-value
Rodent feces in food storage	3.6	1.1-9.8	0.01
Large numbers of field mice near house	2.6	0.6-6.3	0.2
Piped water as water source	2.0	0.1-176.8	0.4
Personal well as water source	1.4	0.3-4.0	0.5
Well protected from rodents	0.3	0.1-1.1	0.04
Eating fresh vegetables	0.2	0.02-0.9	0.02

^a^OR = odds ratio; CI = confidence interval.

**Table 2:** Risk factors for tularemia determined by conditional logistic regression in a matched comparison of 46 case households and 76 control households, Kosovo, October 1999-May 2000

Variable	OR^a^	95% CI	p value
Rodent feces in food storage	5.8	1.5-22.2	0.01
Large numbers of field mice near house	5.7	1.1-28.7	0.04
Well protected from rodents	0.2	0.02-0.99	0.04
Eating fresh vegetables	0.1	0.01-0.80	0.03

^a^OR = odds ratio; CI = confidence interval.

### Environmental Findings

Villages in the affected regions are mostly located in a broad valley with rolling hills, surrounded by steep foothills and mountains in an arc from northwestern to southwestern Kosovo. The Peje Valley has a confluence of rivers from surrounding elevations and is intersected by streams. The environment appeared favorable for the common epizootic hosts of *F. tularensis* in Europe (water voles [*Arvicola* *terrestris*] and common voles [*Microtus* *arvalis*]), but villagers did not describe these rodents, and investigators saw no signs of them. Sites of large colonies of voles were, however, identified in subalpine meadows in the mountains west of the valley, but no trapping was done there. Residents of affected villages readily identified the striped field mouse and the black rat as the animals that had overrun their properties in 1999. In addition, in late summer 1999, villagers noticed unusually large numbers of weasels, which are important rodent predators.

Homes were constructed of cement, stones or bricks, and tile roofs supported by wooden frames; almost all had been severely damaged by fire and explosives and were in various stages of repair. Most were surrounded by yards that included common work areas; small gardens; wells of primitive stone construction with absent or inadequate cement aprons, head cylinders, or protective covers; and outdoor latrines without pits. Rodents had easy access to most wells, where they could find harborage in the rock walls. Livestock and domestic pets were sheltered in crude outbuildings and sometimes on the ground level of residences. Feed corn was stored in cribs with easy rodent access; other animal foodstuffs were typically stored unprotected in open rooms in homes and outbuildings. Fields and gardens were near homes, many were fallow or newly cultivated, and some contained unharvested crops. Household food storage and preparation areas were often poorly protected from rodents and were frequently contaminated by rodent feces.

Sixty-four rodents representing five species were collected: 26 striped field mice (*A. agrarius*); 2 yellow-necked mice (*A. flavicollis*); 2 wood mice (*A. sylvaticus*); 23 house mice (*Mus musculus*); and 11 black rats (*Rattus rattus*). Tissues from these species were tested at the temporary WHO tularemia investigative laboratory in Pristina. *F. tularensis* LPS antigen was detected by antigen-capture ELISA in the liver tissue of *A. agrarius* (Deqan municipality) and in an *A. agrarius* recovered from a well in the village in Gjakova, where the index cases were reported. Of 48 mammalian fecal specimens collected and tested for *F. tularensis* antigen, 5 were antigen positive, 3 from *A. agrarius* and 2 from *R. rattus*.

## Discussion

The clinical picture of fever, pharyngitis, and cervical lymphadenitis, together with serologic confirmation of several hundred cases, indicates that an outbreak of tularemia occurred throughout rural Kosovo during October 1999 to May 2000. The results of the case-control study support the hypothesis that tularemia was foodborne, based on associations of illness with large numbers of rodents in the peridomestic environment, rodent contamination of food storage and preparation areas, and eating some uncooked foods. In addition, epidemiologic and environmental evidence suggested that unprotected and unboiled water contributed to the outbreak. We were not able to explain a possible independent protective value of eating fresh (unprocessed) vegetables, but it may be related to shorter storage and less chance of contamination, a surrogate for better socioeconomic circumstances or less disruption of farming, or an association attributable to chance alone.

This is the first outbreak of tularemia described in Kosovo. The unexpected finding of tularemia immediately after warfare raised questions about the origin of the outbreak and its epidemiologic characteristics. Some authorities expressed concern about possible intentional use of *F. tularensis*, since this pathogen is considered a class A agent for possible bioterrorism use (*11*). However, initial field investigations quickly provided evidence of a widely occurring natural event, most likely resulting from unusual environmental and sociopolitical circumstances in war-torn Kosovo.

The most recent Kosovo census was done in 1981, when reliable population estimates were not available. Since there were no laboratories in Kosovo equipped to work with tularemia, an emergency laboratory was set up to deal with the outbreak samples. Under these circumstances, we performed a matched case-control study of households supported by limited environmental investigations during April to May 2000.

Results of the case-control study were strongly supported by the environmental investigations. The principal populations affected by the tularemia outbreak were ethnic Albanians with limited economic means, living in rural farming villages. These populations had precipitously fled to neighboring areas in the mass exodus during NATO bombing and Serbian reprisals in spring 1999. When refugees returned weeks to months later, they found a vastly disordered environment. Most homes had been ransacked and destroyed by incendiary devices and explosives, food storage areas had been left unprotected, wells had been damaged and contaminated, crops had been left unharvested, and many fields were fallow. Most striking, the returning refugees noted a population explosion of rodents in fields, gardens, homes, and outbuildings. The increased rodent activity in and around homes was reported to have peaked in fall 1999 and continued to be a problem in homes during the winter months. By the time of our investigation, rodent levels had returned to normal. The investigative teams readily found signs of rodent activity in houses and rodent contamination of wells and food materials.

Some rodent tissues and feces sampled contained *F. tularensis* LPS antigen. Although it was considered important to isolate *F. tularensis* from clinical and environmental samples, facilities were not available for growing the organism in culture in Kosovo, and authorities were concerned with the hazards this could pose to laboratory workers.

A disrupted agricultural environment, deserted homes, and unprotected food stores in Kosovo in spring 1999 likely resulted in a rapid increase in rodent populations favorable for epizootic spread of tularemia in rodents and consequent widespread environmental contamination with *F. tularensis*. Although this organism does not produce spores or multiply outside animal hosts, it can survive for months in cold, moist conditions. Large outbreaks of human tularemia in Europe have been described following contamination of the environment with rodent excrement and carcasses (*12*–*18*). The largest occurred as a result of disrupted agricultural environments because of warfare on the Eastern Front during World War II (*12*). These outbreaks have often been associated with a broad spectrum of tularemia syndromes, including high proportions of cases with pleuropneumonic and typhoidal presentations. It is unclear, however, whether these cases arose from inhalation or ingestion exposures, or both. Since we specifically sought cases of glandular or ulceroglandular disease, we may have missed cases with other tularemia syndromes and underestimated the extent of the outbreak. Interviews with patients and their families did not, however, suggest an outbreak of pleuropneumonic or typhoidal tularemia.

Although tularemia has rarely been reported from the Balkans, an outbreak of ulceroglandular tularemia, suspected to be associated with infected hares, was reported in central and western Bosnia in 1995, in the aftermath of warfare (WHO, personal communication). A longitudinal ecologic study of a tularemia natural focus in Croatia revealed that the focus was a meadow-field type and that the common vole was a crucial member of the tularemia biocenosis there (*19*). A report of a large series of cases of tularemia in Turkey, thought to be secondary to drinking of contaminated water, showed that most patients had pharyngitis and cervical adenitis, similar to the cases in Kosovo (*20*,*21*).

Based on the findings of the investigation, general recommendations were made to improve epidemiologic surveillance, provide health education, establish improved water and waste management systems, and strengthen the public health and water and sanitation infrastucture. Educational materials were developed for health professionals and the public that described tularemia, its diagnosis and treatment, and the need for improved sanitation and hygiene, especially rodent control, protection of food and water from rodents and rodent waste, and cooking and boiling food and water. Training and materials were provided to develop a microbiology laboratory capable of diagnosing tularemia. The outbreak highlighted the need for policies that would lead to improved community water sources and waste management throughout Kosovo.
